# Whole-Genome Sequence of Lactobacillus acidophilus PNW3, Isolated from Weaned Piglets of the Indigenous South African Windsnyer Pig Breed

**DOI:** 10.1128/MRA.00362-19

**Published:** 2019-06-20

**Authors:** Kazeem A. Alayande, Olayinka A. Aiyegoro, Collins N. Ateba

**Affiliations:** a Department of Microbiology, North-West University, Mmabatho, South Africa; b Food Security and Safety Niche Area, North-West University, Mafikeng, South Africa; c Agricultural Research Council, Animal Production Institute, Gastrointestinal Microbiology and Biotechnology Division, Irene, South Africa; d Department of Biology, University of Waterloo, Waterloo, Ontario, Canada; University of Arizona

## Abstract

A draft genome sequence of Lactobacillus acidophilus PNW3 is reported. The genome assembly is 1,857,655 bp long in 25 contigs with an *N*_50_ value of 230,557 bp and a G+C content of 34.6%. The total number of predicted protein-coding genes is 1,776, with 58 predicted RNAs and 42 predicted pseudogenes.

## ANNOUNCEMENT

Lactic acid bacteria (LAB) are widely used as probiotics across host species (1). Studies have indicated that bioactive secondary metabolites produced by many probiotic agents affect bacterial community interactions and potentially attenuate disease symptoms caused by pathogens (2, 3).

Lactobacillus acidophilus PNW3 was isolated from the gastrointestinal tract (GIT) of compassionately sacrificed weaned piglets of the indigenous South African Windsnyer pig breed. Each section of the GITs was aseptically transferred into a sterile stomacher bag, and phosphate-buffered saline (PBS) (pH 7) was added before homogenization. This was serially diluted, plated on de Man-Rogosa-Sharpe (MRS) agar, and incubated at 37°C for 48 hours under strict anaerobic conditions. Distinct colonies were streaked on MRS agar for pure cultures and then characterized. The pure culture of L. acidophilus PNW3 was subcultured in de Man-Rogosa-Sharpe broth under complete anaerobic conditions and incubated at 37°C for 24 h in an anaerobic jar provided with the AnaeroGen system (Thermo Fisher Scientific, UK). The broth culture was centrifuged (4,000 rpm for 10 min), and the recovered bacterial cell pellet was washed in PBS. The genome of L. acidophilus PNW3 was extracted using a DNA extraction kit (Zymo Research, USA), and the genomic DNA was prepared with an Illumina Nextera DNA flex library prep kit. This library was sequenced on an Illumina MiSeq instrument at the Agricultural Research Council Biotechnology Platform (Pretoria, South Africa).

A total of 4,944,578 reads were generated with 2 × 300-bp paired-end read lengths. The data were filtered for low-quality reads and adapter regions using Trimmomatic v. 0.32 (4) with a minimum quality score of 15 and a minimum sequence length of 70. The adapter sequences were clipped using a mismatch value of 2, a palindrome clip threshold of 30, and a simple clip threshold of 15. A draft genome was assembled using SPAdes v. 3.12.0 (5) via the KBase platform (6) with a final coverage of 377.0×. The genomic DNA was annotated using the NCBI Prokaryotic Genome Annotation Pipeline (PGAP) v. 4.7 (7, 8) and Rapid Annotations using Subsystems Technology (RAST) with SEED viewer v. 2.0 (9). Rapid *in silico* analysis of secondary metabolite biosynthesis gene clusters was assessed using antiSMASH v. 5.0.0beta1 (10). PathogenFinder v. 1.1 (11) and ResFinder v. 3.1 (12) were used to determine the pathogenicity of L. acidophilus PNW3 toward human hosts and the possible presence of antimicrobial resistance genes. Default parameters were used for all the software employed in the analysis.

The genome assembly size of L. acidophilus PNW3 is 1,857,655 bp, consisting of 25 contigs with a G+C content of 34.6%. Half of the sequence in the entire draft assembly is covered by the contigs of ≥230,557 bp. There are 1,776 annotated protein-coding genes with 55 predicted tRNAs, 3 predicted rRNAs, and 42 predicted pseudogenes. Though the isolate is predicted to not be a human pathogen, lincosamide- and tetracycline-resistant genes were spotted as the only resistant genes harbored by L. acidophilus PNW3. A bioactive protein predicted to be gassericin T was identified as one of the likely secondary metabolites.

All procedures involved in this study complied with relevant legislation regarding protection of animal welfare and were approved by the Agricultural Research Council, API Ethics Committee (APIEC13/008).

### Data availability.

This whole-genome shotgun project has been deposited in DDBJ/ENA/GenBank under the accession number SMLT00000000. The version described in this paper is version SMLT01000000. The SRA accession number is SRX5395058, the BioProject number is PRJNA504734, and the BioSample number is SAMN10979321.
